# Pilot Feasibility Study of Incorporating Whole Person Care Health Coaching Into an Employee Wellness Program

**DOI:** 10.3389/fpubh.2020.570458

**Published:** 2021-03-26

**Authors:** Anna Nelson, Olivia Moses, Brenda Rea, Kelly Morton, Wendy Shih, Fatimah Alramadhan, Pramil N. Singh

**Affiliations:** ^1^School of Public Health, Loma Linda University, Loma Linda, CA, United States; ^2^Risk Management, Loma Linda University, Loma Linda, CA, United States; ^3^Department of Preventive Medicine, Loma Linda University, Loma Linda, CA, United States; ^4^School of Behavioral Health, Loma Linda University, Loma Linda, CA, United States; ^5^Transdisciplinary Tobacco Research Program, Loma Linda University Cancer Center, Loma Linda, CA, United States

**Keywords:** health coaching, corporate wellness, employee health, tobacco, smoking cessation

## Abstract

Prior research supports positive health coaching outcomes, but there is limited literature on the integration of employer-sponsored health coaching into employee wellness strategy. The aim of our mixed methods study was to assess feasibility, acceptability, and preliminary efficacy of incorporating a whole-person care model of health coaching into an employee wellness program (i.e., weight loss, smoking cessation) that is made available by an employer-sponsored health plan. For the quantitative study, eligible employees and covered spouses (*n* = 39) from Loma Linda University Health were recruited into a novel, 12-week, whole person care intervention that combined health coaching and health education and examined outcomes from surveys detailing the participants' experience and biometric data from the intervention and maintenance periods. For the qualitative study, data were collected through key informant interviews from three health coaches and six intervention participants who were recruited *via* random sampling. Health coaching was well-received by the participants, and led to a slight albeit positive behavioral change for obesity. A significant decrease in body mass index occurred over 12 weeks of intervention (−0.36 kg/m^2^, *p* = 0.016), that did not continue during the maintenance phase (−0.17 kg/m^2^, *p* = 0.218). Qualitative findings indicated improved personal health awareness, accountability, motivation, and self-efficacy along with goal setting and barrier overcoming skills among the key themes. Our pilot study findings identify positive behavior change effects of an employee health intervention based on a whole person care model of health coaching with integrated health education, and also identify the need for methods to maintain behavior change (i.e., mHealth, peer-support) post-intervention. Further investigation in randomized controlled trials is the next step in this research.

## Introduction

According to the Centers for Disease Control and Prevention, chronic and mental health conditions accounted for 90% of healthcare costs in the United States in 2016 (1). This represents a continuous steady increase from 75% reported in 2009, and 86% in 2010 (2, 3). In addition to the rising trend of chronic diseases (3), nearly 90% of U.S. physicians report that their patients have social conditions affecting their health (4). Health care systems are recognizing that without addressing the social determinants of health, they may not be able to advance health care beyond the traditional clinical model (5, 6). A recent survey of Medicaid managed care plans found that 91% of the responding plans reported some activities to address social determinants of health (7).

### Health Coaching Success

Health coaching is a relatively new health profession that has formally joined the healthcare system in 2017 and is focused on partnering with patients to “foster healing, optimize health, and enhance well-being” (8). Studies have demonstrated effectiveness of health coaching, especially for patients with chronic conditions (9, 10). In one study looking to increase physical activity through health coaching among pre-diabetics, researchers found that the program was successful after just 12 health coaching sessions. Participants not only increased their physical activity levels, but also improved their food choices. The results were sustained 12 weeks after the end of the intervention (11). In a systematic review of health coaching studies with randomized clinical trial design, 11 articles were identified, and the review concluded that health coaching is effective in lowering weight and increasing healthy food consumption, even among diverse populations (12). Researchers, however, do suggest there remains a gap in the literature evaluating the use of health and wellness coaching among patients who have type 2 diabetes or cancer, are at a high risk for either disease, or have other chronic diseases (10, 12).

Few studies have evaluated whether health and wellness coaching can be incorporated into interventions that address social determinants of health in an employer sponsored health plan, but models that included health coaching and community health workers services helped address the needs of the communities and showed positive improvements in self-reported health, healthcare utilization, and increased confidence managing health issues (13).

### Health Coaching in Employee Health

Employee wellness has emerged as a high impact environment in which to introduce interventions (e.g., weight loss, smoking cessation, and preventive screening) based on social determinants of health. Further incorporation of a health coaching model into such workplace interventions represents a promising next step in employee health and has been tested in a few studies. In one study, where 286 businesses sponsored their overweight or obese employees (*N* = 5,405) to participate in a health coaching program to promote weight loss, the authors found that the intervention was effective and significantly lowered the BMI at 3, 6, and 12 months of follow-up (14). Another study of 7,778 employees found that older employees, females and those in poor health were more likely to participate in coaching activities. Worksite-level and employee-level factors had significant influence on engagement in coaching (15). A more recent example of 2,169 individuals who were enrolled in a health plan of a large health and well-being company, showed that following 6 months of health coaching intervention, there was a significant decrease in a total number of unhealthy days experienced by these employees (16). The limited literature in the field of health and wellness coaching and employee wellness justify further research into the success of integrating a health coaching model in employee health programs.

### Health Plan Model Incentivizing Social Determinants of Health

Loma Linda University Health (LLUH) offers an “opt in” health plan option—the Wholeness Health Plan (WHP)—to its benefit-eligible employees which incentivizes social determinants of health. Through this plan, employees have an opportunity to receive an “opt in wellness discount” on out-of-pocket health plan costs (i.e., monthly premiums, co-pays) (17) by completing specific wellness activities (i.e., interventions such as weight management for high risk patients, smoking cessation for current/relapsed smokers). To date, this innovative workplace health plan model was developed at LLUH and has been used to date to accomplish a high rate of participation (73%) and success (48% 4 months point prevalence abstinence) in WHP sponsored employee smoking cessation (17).

The aim of the mixed methods pilot study in this report was to assess the feasibility, acceptability, and preliminary efficacy of incorporating health coaching into LLUH's innovative WHP that incentivizes improvements in social determinants of health. In addition to efficacy measures in the quantitative study, our goal was to assess acceptability by obtaining during qualitative study interviews both employees' and coaches' perspective of the value of a whole-person health coaching program within a population in a care management program tied to their health plan.

## Methods

This mixed methods study was designed to include: (1) a quantitative study (one-arm intervention) of the efficacy of the health coaching intervention on improving metabolic panel outcomes and anthropometric outcomes; and (2) a qualitative study (key informant interviews) on assessing feasibility and acceptability of the health coaching intervention among participants and coaches. These are described below.

### Quantitative Study for Assessment of Efficacy

The quantitative study of employee participants was designed to be a one arm uncontrolled intervention study. Consented employees were enrolled in the 12 weeks intervention phase and 12 weeks maintenance phase.

#### Participant Recruitment

To be eligible for the study, Wholeness Health Plan (WHP) members had to meet the following three inclusion criteria: (a) blood pressure ≥ 130/80 mm Hg; (b) fasting blood sugar ≥ 100 mg/dl OR non-fasting blood sugar ≥ 140 mg/dl, and (c) total cholesterol ≥150 mg/dl OR LDL-C ≥ 130 mg/dl OR triglycerides ≥150 mg/dl OR HDL <40 mg/dl. Eligible employees identified during the required Wholeness Health Plan wellness discount's biometric screening received an invitation letter from the Health Plan to visit their physician. Upon completion of the physician's appointment they were invited to join the study that used the health coaching methods described below. Since health coaching was being tested for inclusion in the incentivized health plan model that has over a 90% participation rate, we are not considering participation rate as a feasibility outcome. The participation rate for the present study (i.e., outside of health plan incentive model) was about 5% and consistent with other voluntary wellness programs that LLUH has run in previous years. Of the 50 subjects enrolled in health coaching, 11 dropped out during the follow-up (22% dropout).

#### Health Coaching Intervention

The health coaching program consisted of 12 weekly 30-min phone sessions and was added to the medical standard of care. Additionally, participants received an initial foundation session of 45 min. The first 4 weeks of the program included a health education focus covering the topics of hypertension, dyslipidemia, diabetes, and weight management. These topics were customized to the participants based on their biometric screening, lab results, and the pre-program survey. The remaining 8 weeks of the program utilized standard models of health coaching combined with LLUH's approach to whole person care. Services were provided by certified health coaches. The program provided comprehensive coaching and tools focused on empowering members to make healthy lifestyle choices that may prevent, control or reverse their conditions. The detailed schedule of intervention activities is listed in the **Appendix**.

Coaches and participants contacted each other *via* email, telephone, and electronic conferencing; additional contacts occurred between scheduled coaching sessions as needed. Participants were encouraged to explore barriers to change and the need for spiritual support while receiving up-to-date evidence-based health information on lifestyle and chronic disease during the 12 weeks of active intervention.

#### Data Collection and Analysis for Assessment of Preliminary Efficacy

Participants were administered surveys at baseline and 12 weeks. Diagnostic laboratory panels (creatinine, HbA1c, a full lipid panel, and comprehensive metabolic panel) and anthropometrics [body mass index, body fat% (TANITA scale)] were administered to participants at baseline, 6, 12, 18, and 24 weeks. A research physician monitored temporal changes in these wellness measures and provided feedback to patients.

Thus, surveys and lab panels during the first 12 weeks were used to assess pre-/post preliminary efficacy. A maintenance effect was assessed at week 18 and 24 weeks. To assess a preliminary pre/post effect we compared survey and lab data between baseline and 12 weeks using generalized linear models for repeated measures to compute contrasts for continuous variables from the lab panels and anthropometrics. The same method was used to assess a maintenance effect but here the contrasts of interest were as follows: (1) baseline to 24 weeks; and (2) post intervention 12–24 weeks.

### Qualitative Study for Assessment of Feasibility and Acceptability

The qualitative study included key informant interviews with health coaches and participants to assess the feasibility and acceptability of the whole person care health coaching intervention.

#### Participant Recruitment

In order to gain additional insight into the feasibility and acceptability of the health coaching intervention, participants were randomly selected from the quantitative study and the first six who consented to a further interview were enrolled. Additionally, all health coaches in the program (*n* = 3) were invited and consented to participate in the key informant (KI) interviews upon completion of the intervention.

#### Key Informant Interview Methods

The interviews were used to assess the perceived impact of the intervention on outcomes as well as to gain perspective from the participants and providers on the strengths and gaps of the health coaching model used. All interviews were completed during October-November, 2019. Six participants were approached by the health coaches with an invitation to participate in the key informant interviews. All of the approached participants provided written consents, and telephone appointments for the interviews were scheduled. The three health coaches in the program were also approached by the investigators, and consented to participate in the KI interviews. All of the interviews followed a KI guide developed specifically for this study. The interviews lasted between 40 and 60 min each and were audio recorded with participants' consent.

#### Qualitative Data Analysis

The audio transcripts were transcribed, and then coded using NVivo Version 12 Pro (QSR International). Thematic analysis was used to determine the key semantic themes in the dataset. We used an inductive approach in which the analysis process was data driven—themes were identified based on the data rather than pre-existing codes. After reviewing the dataset, we generated the initial codes from the data organizing quotes into meaningful categories based on the patterns, and then, organized these into emergent themes.

## Results

Findings from the mixed method study are summarized from the quantitative assessment (*n* = 39 enrolled in a one arm intervention) and the qualitative study (Interviews of six participants, Interviews of three coaches).

### Quantitative Assessment of Preliminary Efficacy

**Figure 1:**
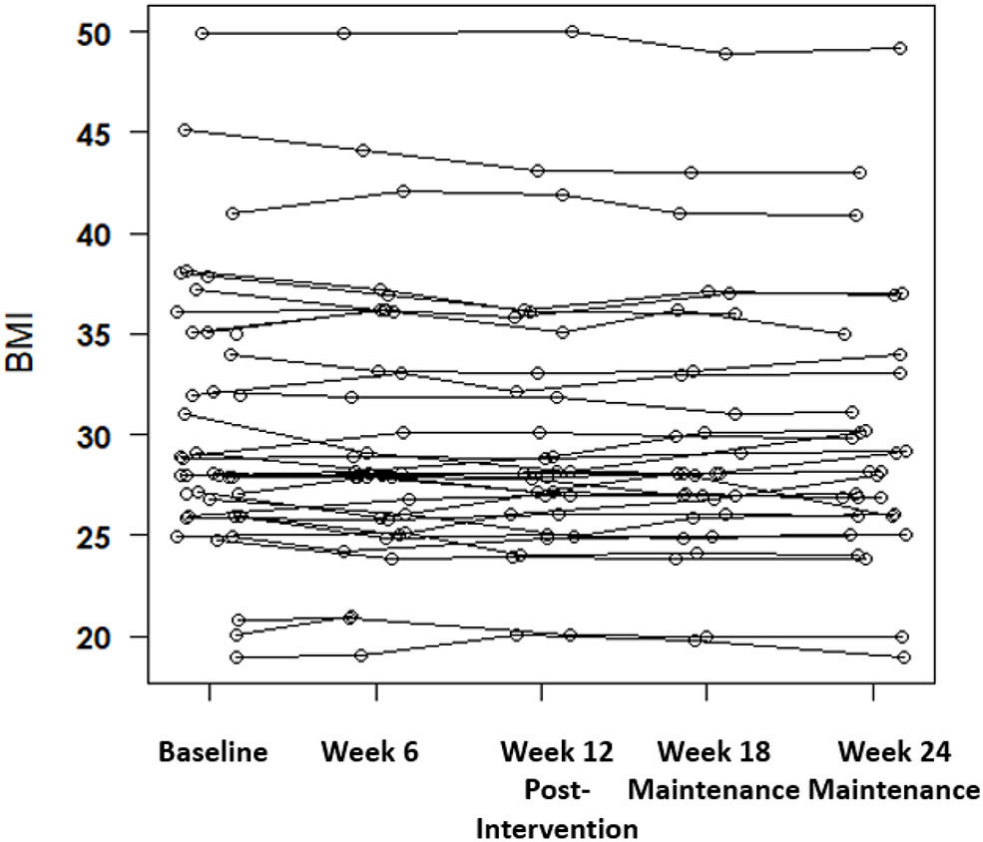
Intervention and maintenance phase BMI measures for a one-arm health coaching intervention with a significant decrease in BMI from baseline to post intervention (*p* = 0.016).

Table 1 provides demographic information as well as the biometric profile of the participants in the study. To determine preliminary efficacy of health coaching (12 weeks coaching + 12 weeks maintenance) in a one arm uncontrolled study sample, we tested intervention contrasts [baseline to post intervention (week 12)], and maintenance contrasts [baseline to end of follow-up (week 24)], post intervention (week 12) to end of follow-up (week 24) across the biometric measures. For body fat, creatinine, HbA1c, and total cholesterol we found no significant or biologically important contrasts based on intervention and/or maintenance. For BMI we found a significant decrease in BMI (Figure 1) from baseline to post intervention (−0.36 kg/m^2^, *p* = 0.016) that did not remain after maintenance (−0.17 kg/m^2^, *p* = 0.22).

**Table 1:** Demographic and biometric profile of the intervention sample (*n* = 39).

**Demographic variable**	
Age (Mean [*SD*])	51.74 [10.96]
Female gender (%)	64%
Ethnicity	
Hispanic or Latino	18%
Not hispanic or Latino	82%
**Baseline biometrics variables (Mean [*SD*])**	
Body mass index (kg/m^2^)	30.54 [6.53]
Body fat (kg)	36.12 [9.50]
Creatinine	0.85 [0.18]
HbA1c	6.04 [1.16]
Total cholesterol	208.34 [59.29]

### Qualitative Assessment of Acceptability

Key Informant interviews were conducted with six Wholeness Health Plan members participating in the study and three health coaches (HC), who led the coaching sessions for this group. Participants mean age was 50 years old (SD = 9.4), four of the plan member participants were female, and five were non-Hispanic/Latino.

Based on the thematic analysis, the value of health coaching was identified in five key themes expressed by the participants and validated by the health coaches: (1) increased personal health awareness and motivation; (2) goal setting and accountability; (3) self-efficacy; (4) value of individualized support; and (5) value of employer-offered coaching.

#### Increased Personal Health Awareness and Motivation

All of the participants expressed that the health coaching program improved their personal health awareness, as well as increased their health knowledge: “It basically opened my eyes to things I was not fully aware of.” “It just helped me take the time to look at areas that I wanted to improve in.” A number of participants remarked of the awareness and motivational value of the frequent lab work combined with coach-led health education: “It helped me by learning that my levels were high, so I need to get it down. That is what helped me with the diet.”

Additionally, several participants mentioned the value of the digital apps recommended by their health coaches: “I was able to download apps and stuff that was able to help me work out.” Yet, another participant appreciated the use of MyFitnessPal for making better dietary choices: “During the study I was using MyFitnessPal app where I was just documenting everything I ate and the quantities. And that helped me identify, like, these foods that I thought were healthy… but it turns out that, um, every time I punch that into the app, it's like, okay, that has a gram of sodium in it.”

The health coaches validated these findings expressing that health coaching sessions allowed the participants to focus on personal health: “Some people were just putting some of their goals on the back burner. It was something they knew they had to do, but it didn't seem real, until they were really talking about it and then emphasizing how important it was.”

#### Goal Setting and Accountability

Five of the participants spoke of the value of the skills they learned through health coaching, in setting goals and overcoming barriers: “It really helped, like, setting up my own goals… and slowly reaching that plan we set up.” “I continued to improve in my goal setting abilities… If my coach wasn't constantly asking me what goals I wanted to achieve, I wouldn't have necessarily thought about all the different factors that ended up being addressed during the time we were working on them.” The health coaches validated these findings by sharing that the goal setting process was among the most helpful factors of health coaching for their clients: “This particular person found this very practical and not overwhelming. Being able to, to have something on a weekly basis that would challenge themselves to meet that overall goal they're working on. So those were helpful in terms of helping them with just over all aspects of lifestyle.”

As part of the goal setting, participants mentioned the value receiving guidance on how to overcome potential barriers. For example, one participant stated: “When we were approaching Thanksgiving holiday, and I was actually really concerned… that it's the beginning of the end because, you know, Halloween and then Thanksgiving and then Christmas. They would say for this party, bring um, your own healthy alternative. If you're at Thanksgiving dinner, have your first serving and then wait 10 min before you have a second serving. Like very, very specific, not vague like ‘oh just try to be healthy.’ Which, I thought was really, really practical.”

Along with goal setting, the theme of accountability was present in all but one of the participant interviews. The weekly contact with the health coaches resulted in accountability ensuring participants made better health choices throughout the week: “I needed to be attentive, I am doing this study, and I need to either lie through my teeth and say I am going to do it, or I need to get it done… I need to be accountable.” “Knowing that next week she is going to call me and ask me how I did… Knowing that in the back of my head, I am, like, okay, I should do better this week, because I don't want to have to tell her that I did bad.” This theme was validated by all health coach interviews.

#### Self-Efficacy

Through learning of the goal setting skills and planning for potential barriers, the participants felt more confident about making changes to their lifestyle and achieving the desired outcomes. One participant stated, “It helped me realize that it's possible. That something can be done. I get home and my family has this food on the table, and that's the only thing I am going to eat, but I have the confidence that if I am on top of things, if I plan things out in advance, if I am prepared, I can do it.”

All three of the coaches validated the increase in confidence among the participants: “I can think of that particular participant that was struggling with eating a lot of fast food, his confidence increasing with being more comfortable in the kitchen and, I think, in part that was due to some of the encouragement, you know, getting that crock pot out and experimenting with recipes and new cookbooks; and I think just the accountability or the feedback they received was very helpful in increasing that confidence that they can achieve their goals.”

Another coach mentioned: “Seeing the actual changes, in their lab work was also reassuring for them and boosted their confidence. Some of these people had not seen these types of changes, ever. You know, they never saw their numbers begin to go the opposite direction. Whether it was weight, or cholesterol, A1C. So it was just reaffirming that what they were doing was positive because it was actually causing changes in their, in their numbers and also in the way that they were just feeling overall.”

#### Value of Individualized Support

Three of the participants spoke specifically of the value of health coaching as encouraging personal support and useful resources: “Having the personal connection with somebody is a huge factor for me. I'm not a book learner. I don't go on the internet to learn a lot so to have somebody calling and touching base and a human voice attached to it was a huge success factor for me.” The value of the encouraging support was underscored by another participant: “HC was not judgmental. HC was encouraging. HC taught me to forgive myself if I didn't make my goals, not be so hard on myself. You know what else? HC shared with me sometimes own personal struggles to be relatable and I appreciated that. It just really showed me how much connecting with people and accountability make a difference in my health.” Another participant stated: “I think they were on point because, we discussed personally my personal needs vs. you know in general, kind of like, what I needed to work on myself, you know, so it wasn't just like a, like a doctor's appointment kind of thing.”

This was validated in one of the coach interviews: “In the health coaching itself there's a lot of affirmation. So for example, you know, um we kind of praise them when they do something positive… And the same can be said for the other thing too. When there was a kind of a lapse, like a relapse in their behavior or in their numbers, just being there to provide support and say hey you know this isn't the end of the world, we can just do these things different and kind of go back to the drawing board.”

#### Value of Employer-Offered Health Coaching

All participants and coaches expressed that employer-sponsored health and wellness coaching programs would be valuable to them. Two of the participants specifically mentioned the organization's overall health and wholeness focus as well as the Blue Zone connection. All participants expressed hope that the health coaching programs could be expanded further: “I hope that it ends up being something they offer to employees.” “I think it should be wide spread. I think it should be part of the insurance coverage.”

All of the health coach interviews validated this theme, with one adding that it is important that people do not feel obligated to participate in health coaching: “It just depends how it's pitched. You know, because as a health coach I think when somebody's kind of required to do something… sometimes they don't, their motivation isn't as high. They're being forced to do something…, so the motivation isn't really there. They're just kind of jumping through the hoops basically… I think, you just get more results and it's a better experience for the health coach and for the participant when the person really wants to be there rather than they're being forced to be there.”

## Discussion

The focus of our mixed methods study was to determine the preliminary efficacy, feasibility, and acceptability of implementing a whole-person health coaching program by conducting a one-arm intervention and a set of interviews (participants, coaches) within a population in a care management program tied to their health plan. In the quantitative assessment, preliminary efficacy of the intervention was shown for obesity with a significant decrease in BMI being evident at 12 weeks post intervention, but attenuating at 24 weeks (maintenance). The intervention also provided feasibility outcomes indicating a 22% dropout rate during health coaching. We note that health coaching was being tested for inclusion in Wholeness Health Plan that currently has a >90% “opt in” participation rate that supports feasibility. Our qualitative study provided interviews indicating a high level of participant and coaches acceptability of whole person health coaching as a valuable intervention model to improve health.

### Acceptability of Whole Person Health Coaching

Some of the reasons for high acceptability included the value of health coaching in raising personal health awareness and the resulting engagement and motivation to improve health, specifically referencing the value of the frequent lab work. This follows the current literature suggesting biometric screening promotes individual awareness and understanding of the results (18). Based on the qualitative data, the combination of regular biometric screening, health coaching, and coach-led health education resulted in the participants becoming better aware of their health status and identifying goals for improvement.

Literature also confirms that the inclusion of the common key features that were a part of our intervention: goal setting, motivational interviewing, and collaboration with primary care providers do increase the effectiveness of the health coaching programs (19). Raising client's accountability is a key outcome of interactions between the client and the coach as was also seen among our participants (20). It has been suggested that including aspects of accountability in healthcare may improve the adherence to the outlined healthcare plan and help reach personalized health goals (21–23). Additional studies point out the value of combining accountability with real-time feedback, which is exactly how health coaching works (24, 25).

Furthermore, planned or responsive adaptations or assisting clients with potential barriers as they engage in behavioral changes as was done in this intervention has been seen as effective tools for overcoming these barriers in prior studies and is associated with enhanced motivation and self-efficacy (26).

Both participants and coaches were satisfied with the frequency of their coaching sessions and only expressed a wish that these sessions would continue beyond the 12-week cut off. A systematic review of 41 health coaching trials suggests there is no current evidence of a dose response effect on the biomarker or health behavior outcomes (20). Our quantitative data, however, present evidence of the behavioral decay following the completion of the coaching program, suggesting that the health coaching effect may dissipate during the maintenance stage possibly due to decreased accountability. The undetermined cost effectiveness of health coaching may be a potential barrier preventing integration of long-term health coaching into health plans. In a review of 27 studies relating to health coaching and costs, Hale and Giese (27) found that while health coaching has been found effective for chronic disease management, the literature was inconclusive whether it lowered health cost expenditures; however, suggested potential long-term future savings.

While it may be financially challenging to offer on-going health coaching, one way coaching interventions may be sustainable is if participants became involved in “peer-coaching” becoming accountable to each other at the end of the professional health coaching intervention. The approach of social accountability was found to improve effectiveness of health interventions in the past (24). A similar component has been found successful in 12-Step Programs. In the study of outcomes among Alcoholic Anonymous, Witbrodt et al. (28) found that those participants who maintained a regular or even somewhat regular connection with their sponsor, had better abstinence outcomes than those who did not. Future health coaching studies could explore whether such “sponsor-model” utilizing “peer-coaching” would be effective in maintaining health behaviors after the completion of the professional health coaching intervention.

Furthermore, health and wellness coaching efforts could be complemented by the utilization of smartphone-based virtual health coaching which has demonstrated positive effects in recent studies (29–33). At the completion of the live health coaching intervention, digital health coaching could take over, providing the lower-cost self-management tools and accountability which would help individuals to remain on track. Similar models exist, and the combination of live, electronic and peer coaching should be researched further (30).

### Limitations

The study's uncontrolled design and small sample size limits the inferences that could be made about the quantitative assessment of the preliminary efficacy of the intervention.

## Conclusions

Our study suggests that incorporation of a whole-person care health coaching component into an employee wellness program may receive positive reception by the interested employees, and can result in positive behavioral changes, as well as statistically significant decreases in certain biomarkers. We did find that this effect dissipated during the maintenance stage. Future trial studies utilizing larger sample sizes and combining peer-coaching with digital health coaching as part of the follow-up to the initial health coaching intervention should be explored before finalizing the coaching model to be integrated in an employee wellness program.

## Data Availability Statement

The raw data supporting the conclusions of this article will be made available by the authors, without undue reservation.

## Ethics Statement

The studies involving human participants were reviewed and approved by Institutional Review Board of Loma Linda University. The patients/participants provided their written informed consent to participate in this study.

## Author Contributions

AN wrote the report and conducted the qualitative research data analysis. OM conceptualized the study, directed the study, collection of data, and edited the report. BR provided clinical oversight of the study and edited the report. KM worked on study design and edited the report. WS analyzed quantitative data for the report and edited the report. PS collaborated on conceptualizing the study, designing the study, obtaining funding, and editing and writing the report. FA contributed to the writing of the report. All authors contributed to the article and approved the submitted version.

## Conflict of Interest

The authors declare that the research was conducted in the absence of any commercial or financial relationships that could be construed as a potential conflict of interest.

## Appendix

### Health Coaching Intervention Schedule

**Table:**

**Week**	**Health coaching modules**
1	**Introduction of coach and client and program review**. A review of biometric screening numbers will be done to determine areas which the client is willing to work on depending on the condition(s) present. The session will collaboratively identify goals and strategies to support goals. The session will help the client identify barriers to reaching and maintain health goals. The final part of the session will consist of a summary of what was covered, and a discussion of the tasks participants will do during the next week. The session (an each thereafter) will end with arranging for a follow-up appointment
2	**Review basics of a healthy diet**. After the review of dietary intervention methods, the session will end with a summary of what was covered, and a discussion of the tasks participants will do during the next week.
3	**Review the benefits of exercise**. The session reviews the basic physical activity recommendations. The final part of the session consists of a summary of what was covered, and a discussion of the tasks participants will do during the next week.
4	**Review the importance of stress management and sleep**. This session teaches participants how to reduce and deal with stress. The final part of the session consists of a summary of what was covered, and a discussion of the tasks participants will do during the next week.
5–11	**Review progress of health goals**. These sessions are client centered and less didactic and/or prescriptive in nature. The coach meets the client at their point of need and concern and follows the interests and needs of the client. The sessions are designed to motivate participants to go forward with their goals.
12	**Review overall progress of health goals**. Finalize action plan.

## References

[1] National Center for Health Statistics Health United States in Health United States. 2016: With Chartbook on Long-term Trends in Health. Hyattsville, MD: National Center for Health Statistics (US). (2017). 28910066

[2] Blackwell J Collins M Scribner C Guillen J Moses K Gregory-Mercado K. Health and wellness coaching implemented by trainees: impact in worksite wellness. Glob Adv Health Med. (2019) 8:2164956119831226. 10.1177/2164956119831226 30834178 PMC6393823

[3] Raghupathi W Raghupathi V. An empirical study of chronic diseases in the United States: a visual analytics approach to public health. Int J Environ Res Public Health. (2018) 15:431. 10.3390/ijerph15030431 PMC5876976 29494555

[4] Norbeck T. Survey of America's Physicians: Practice Patterns and Perspectives. Merritt Hawkins, on behalf of the Physicians Foundation. (2018).

[5] Houlihan J Leffler S. Assessing and addressing social determinants of health: a key competency for succeeding in value-based care. Primary Care. (2019) 46:561–74. 10.1016/j.pop.2019.07.013 31655752

[6] Thomas KS Durfey SN Gadbois EA Meyers DJ Brazier JF McCreedy EM. Perspectives of Medicare Advantage plan representatives on addressing social determinants of health in response to the CHRONIC care act. JAMA Netw Open. (2019) 2:e196923. 10.1001/jamanetworkopen.2019.6923 31298711 PMC6628593

[7] Artiga S Hinton E. Beyond health care: the role of social determinants in promoting health and health equity. Health. (2019) 20:10. Available online at: https://www.kff.org/racial-equity-and-health-policy/issue-brief/beyond-health-care-the-role-of-social-determinants-in-promoting-health-and-health-equity/

[8] Kreisberg J Marra R. Board-certified health coaches? what integrative physicians need to know. Integr Med. (2017) 16:22. 30936812 PMC6438087

[9] Boehmer KR Barakat S Ahn S Prokop LJ Erwin PJ Murad MH. Health coaching interventions for persons with chronic conditions: a systematic review and meta-analysis protocol. Syst Rev. (2016) 5:146. 10.1186/s13643-016-0316-3 27585627 PMC5009492

[10] Sforzo GA Kaye MP Todorova I Harenberg S Costello K Cobus-Kuo L. Compendium of the health and wellness coaching literature. Am J Lifestyle Med. (2018) 12:436–47. 10.1177/1559827617708562 30542254 PMC6236633

[11] DeJesus RS Clark MM Rutten LJF Jacobson RM Croghan IT Wilson PM. Impact of a 12-week wellness coaching on self-care behaviors among primary care adult patients with prediabetes. Prev Med Rep. (2018) 10:100–5. 10.1016/j.pmedr.2018.02.012 29850394 PMC5966585

[12] Kennel J. Health and wellness coaching improves weight and nutrition behaviors. Am J Lifestyle Med. (2018) 12:448–50. 10.1177/1559827618792846 30783395 PMC6367877

[13] Russell D Oberlink MR Shah S Evans L Bassuk K. Addressing the health and wellness needs of vulnerable rockaway residents in the wake of hurricane sandy: findings from a health coaching and community health worker program. J Public Health Manag Practice. (2018) 24:137–45. 10.1097/PHH.0000000000000545 28257399

[14] Merrill R Aldana S Bowden D. Employee weight management through health coaching. Eating Weight Disord. (2010) 15:e52–9. 10.1007/BF03325280 20571321

[15] Grossmeier J. The influence of worksite and employee variables on employee engagement in telephonic health coaching programs: a retrospective multivariate analysis. Am J Health Promotion. (2013) 27:e69–80. 10.4278/ajhp.100615-QUAN-190 23286600

[16] Cole S Zbikowski SM Renda A Wallace A Dobbins JM Bogard M. Examining changes in healthy days after health coaching. Am J Health Promotion. (2019) 33:774–7. 10.1177/0890117118816286 30497272

[17] Moses O Rea B Medina E Estevez D Gaio J Hubbard M. Participation in a workplace smoking cessation program incentivized by lowering the cost of health care coverage: findings from the LLUH BREATHE cohort. Tobacco Prev Cessation. (2020) 6:118237. 10.18332/tpc/118237 32548360 PMC7291893

[18] Kaufman HW Williams FR Odeh MA. Value of laboratory tests in employer-sponsored health risk assessments for newly identifying health conditions: analysis of 52,270 participants. PLoS ONE. (2011) 6:28201. 10.1371/journal.pone.0028201 22163283 PMC3233567

[19] Olsen JM Nesbitt BJ. Health coaching to improve healthy lifestyle behaviors: an integrative review. Am J Health Promotion. (2010) 25:e1–12. 10.4278/ajhp.090313-LIT-101 20809820

[20] Gierisch JM Hughes JM Edelman D Bosworth HB Oddone EZ Taylor SS. The Effectiveness of Health Coaching. Washington, DC: Department of Veterans Affairs (US) (2017). 29553632

[21] Hardcastle SJ Glassey R Salfinger S Tan J Cohen P. Factors influencing participation in health behaviors in endometrial cancer survivors. Psycho-oncology. (2017) 26:1099–104. 10.1002/pon.4288 27665487

[22] Oussedik E Cline A Su JJ Masicampo EJ Kammrath LK Ip E. Accountability in patient adherence. Patient Preference Adherence. (2019) 13:1511. 10.2147/PPA.S213113 31564838 PMC6732501

[23] Oussedik E Foy CG Masicampo EJ Kammrath LK Anderson RE Feldman SR. Accountability: a missing construct in models of adherence behavior and in clinical practice. Patient Preference Adherence. (2017) 11:1285. 10.2147/PPA.S135895 28794618 PMC5536091

[24] Forster HA Zaval L Jachimowicz J Berry D Weber EU. Friends with benefits: social accountability increases physical activity. PsyarXiv [Preprint]. (2020). 10.31234/osf.io/dkce2

[25] Stubbins R He T Yu X Puppala M Ezeana CF Chen S. A behavior-modification, clinical-grade mobile application to improve breast cancer survivors' accountability and health outcomes. JCO Clin Cancer Inform. (2018) 2:1–11. 10.1200/CCI.18.00054 PMC10445791 30652617

[26] Betts AC Froehlich-Grobe K Driver S Carlton D Kramer MK. Reducing barriers to healthy weight: planned and responsive adaptations to a lifestyle intervention to serve people with impaired mobility. Disabil Health J. (2018) 11:315–23. 10.1016/j.dhjo.2017.10.008 29129715 PMC5869071

[27] Hale R Giese J. Cost-effectiveness of health coaching. Professional Case Manag. (2017) 22:228–38. 10.1097/NCM.0000000000000223 28777235

[28] Witbrodt J Kaskutas L Bond J Delucchi K. Does sponsorship improve outcomes above Alcoholics Anonymous attendance? A latent class growth curve analysis. Addiction. (2012) 107:301–11. 10.1111/j.1360-0443.2011.03570.x 21752145 PMC3260344

[29] Everett E Kane B Yoo A Dobs A Mathioudakis N. A novel approach for fully automated, personalized health coaching for adults with prediabetes: pilot clinical trial. J Med Internet Res. (2018) 20:e72. 10.2196/jmir.9723 29487046 PMC5849796

[30] Kowitt SD Tang PY Peeples M Duni J Peskin S Fisher EB. Combining the high tech with the soft touch: population health management using eHealth and peer support. Population Health Manag. (2017) 20:3–5. 10.1089/pop.2016.0027 27267800

[31] Mao AY Chen C Magana C Barajas KC Olayiwola JN. A mobile phone-based health coaching intervention for weight loss and blood pressure reduction in a national payer population: a retrospective study. JMIR mHealth uHealth. (2017) 5:e80. 10.2196/mhealth.7591 28596147 PMC5481661

[32] Wayne N Ritvo P. Smartphone-enabled health coach intervention for people with diabetes from a modest socioeconomic strata community: single-arm longitudinal feasibility study. J Med Internet Res. (2014) 16:e149. 10.2196/jmir.3180 24907918 PMC4071226

[33] Willey S Walsh JK. Outcomes of a mobile health coaching platform: 12-week results of a single-arm longitudinal study. JMIR mHealth uHealth. (2016) 4:e3. 10.2196/mhealth.4933 26747611 PMC4723727

